# Microwave-assisted synthesis of biodiesel by a green carbon-based heterogeneous catalyst derived from areca nut husk by one-pot hydrothermal carbonization

**DOI:** 10.1038/s41598-022-25877-w

**Published:** 2022-12-12

**Authors:** Gaurav Yadav, Nidhi Yadav, Md. Ahmaruzzaman

**Affiliations:** Department of Chemistry, National Institute of Technology, Silchar, Assam 788010 India

**Keywords:** Catalyst synthesis, Heterogeneous catalysis

## Abstract

In this study, we have synthesized a solid acid catalyst by areca nut husk using low temperature hydrothermal carbonization method. The fabricated catalyst has enhanced sulfonic actives sites (3.12%) and high acid density (1.88 mmol g^−1^) due to –SO_3_H, which are used significantly for effective biodiesel synthesis at low temperatures. The chemical composition and morphology of the catalyst is determined by various techniques, such as Fourier transform infrared (FTIR), powder X-ray diffraction (XRD), Brunauer–Emmett–Teller (BET), Scanning electron microscope (SEM), Energy disruptive spectroscopy (EDS), Mapping, Thermogravimetric analysis (TGA), CHNS analyzer, Transmission electron microscopy (TEM), particle size analyzer, and X-ray photoelectron spectroscopy (XPS). Acid–base back titration method was used to determine the acid density of the synthesized material. In the presence of the as-fabricated catalyst, the conversion of oleic acid (OA) to methyl oleate reached 96.4% in 60 min under optimized conditions (1:25 Oleic acid: methanol ratio, 80 °C, 60 min, 9 wt% catalyst dosage) and observed low activation energy of 45.377 kJ mol^−1^. The presence of the porous structure and sulfonic groups of the catalyst contributes to the high activity of the catalyst. The biodiesel synthesis was confirmed by gas-chromatography mass spectrometer (GC–MS) and Nuclear magnetic resonance (NMR). The reusability of the catalyst was examined up to four consecutive cycles, yielding a high 85% transformation of OA to methyl oleate on the fourth catalytic cycle.

## Introduction

Sustainable development refers to effective methods for meeting current energy demand while using natural assets and preserving them for succeeding generations^1^. Through manufacturing enterprises and government agencies, the scientific and technical civilization has been maximized in encouraging housing, materials, clean energy, food safety, and even urban planning research initiatives^2^. Even though a substantial study has previously been conducted on energy exploration of crude oil deposits with improved oil recovery methods, the economics of its utilization using present technology remain very questionable. By 2040, the world population is predicted to grow by 50%, resulting in increased energy consumption. However, to alleviate current climate change and CO_2_ emissions, the gap betwixt power demand and supply must be narrowed by deploying renewable sources^3,4^.

Biodiesel or FAME (fatty acid methyl ester) is a biodegradable, renewable, non-toxic, and CO_2_-neutral energy source. Biodiesel combustion characteristics are incredibly similar to petroleum diesel^5^. Similar physical and chemical characteristics suggest that biodiesel may be utilized in diesel engines without requiring engine modification. Due to its incredible potential as a valuable energy source in the future, several researchers have worked to use biodiesel as a sustainable energy source^6^. Biodiesel production frequently makes use of both heterogeneous and homogeneous catalysts. There are various disadvantages to using homogeneous catalysts due to highly sensitivity toward water and free fatty acid^7^. Along with this, homogeneous catalysts lead to soap accumulation due to side reaction. As a result, increased focus has been paid to the burgeoning of solid-phase esterification catalysts. Several acidic and basic catalysts, including mixed oxides, transition metal oxides, metal oxides, hydrotalcite, ion exchange resin, zeolites, and carbon-based, are available for biodiesel formation. Fuel made from algae biomass, non-edible plants, animal fats, and waste oils is being touted as a viable alternative or addition to traditional diesel^5,6^. Heterogeneous catalysts derived from chemicals also have several disadvantages like leaching, microporosity, fewer active sites, toxicity, and environment-unfriendliness^8,9^. On the other hand, heterogeneous catalysts often cause expensive and complex fabrication methods such as numerous steps and high temperatures or have not utilized biogenic resources. Catalysts derived from low-cost biomass for biodiesel production are used to replace conventional fossil fuels, making them an attractive alternative^10^. As a result, scientists are now compelled to develop biodiesel from sustainable biomass and non-edible sources. Carbon-based materials gain interest due to sustainability, cost-efficient, economic, and renewable resources. Carbonaceous materials have significant applications such as catalytic fuel production^11^, energy storage^12^, and carbon aerogels^13^. The allure of utilizing biomass in this manner stems from the possibility of lowering manufacturing production by reusing generally sustainable, non-toxic natural resources. Heterogeneous catalyst derived from biomass offers environment-friendly alternatives since they are non-toxic, non-corrosive, don't generate secondary waste, and are easily separable from the reaction mixture. Moreover, the biodegradation of catalyst does not cause concern because of the disposal challenge. The effective fabricated catalyst made from biomass has a large surface area and wide pore diameter^14^.

Hydrothermal carbonization encourages carbonaceous materials production at low temperatures; it has become increasingly appealing^15^. This method of producing carbonaceous materials from naturally existing biomass has coupled the utilization of renewable sources with appealing porosity and stability. Due to these combined benefits, researchers are looking at ways to functionalize cyclic carbon compounds with active catalytic Bronsted acid groups (usually –SO_3_H)^16^. Low sulfur content and inactivity are commonly characteristic of carbon materials derived from unprocessed plant material^17^. These two factors prompted us to investigate biomass utilizing a one-pot, low-temperature procedure as a sulfonic acid-carbon precursor.

Biodiesel synthesis by microwave-assisted techniques has been recently tested. In the microwave technique, the temperature of the reaction mixture reaches in shorter duration. The microwave technique also bypasses the wall effect and provides direct energy via the reaction mixture, lowering total energy consumption^18^. Microwave-assisted fabrication of biodiesel may speed up by microwave radiation. Carbon materials are excellent for absorbing microwave radiation giving rise to temperature. Carbon-based catalyst is a material with stable physical properties and a large surface area. Because of this, it might serve as the catalyst's carrier in a microwave reaction. In addition, the polar Bronsted acid sulfuric acid, which is placed onto carbon, has a strong ability to absorb microwave radiation^19^.

*Areca nut husk *belongs to the Arecaceae family. *Areca nut husk* or betel nut, or supari, is largely found in India. The identification of areca nut husk is made from the literature^20^. The husk of areca nut is waste material and has no use. In Southeast Asia, the areca nut is one of the most significant fruits for economic purposes. This tropical fruit provides a rich source of protein, fibers, polyphenols (a kind of antioxidant), and fatty polysaccharides. *Areca nut husk* is an ingredient of pan masala and is commercially utilized in leather tanning and food coloring. India ranked 1st among the producer of areca nut, followed by Bangladesh, China, and Indonesia. Around 224 tonnes of *Areca nut husk* are produced in India each year, accounting for approximately 20% to 35% of the raw areca nut. *Areca nut husk* is so plentiful that it is typically discounted as insignificant and disposed of in an ineffective or wasteful manner.

The present study describes the one-pot processing of *Areca nut husk* into a biogenic, inexpensive, and recyclable material. The catalyst is prepared via the one-pot hydrothermal carbonization method, which is eco-friendly. Generally, the temperature range of catalyst formation is 120–260 °C used during hydrothermal carbonization. However, in this work, authors have tried to form hydrochar at a low temperature (80 °C) with the insertion of active sulfonic sites. These concepts have shifted emphasis to the possibility of thermochemically digesting plant biomass to produce carbonaceous materials. These advancements promise to make catalyst manufacturing more affordable and efficient by including SO_3_H groups while preserving slightly acidic hydrophilic molecules capable of sequestering water and promoting esterification. The application of sulfonated *Areca nut husk* (SANH) for esterification reaction has not been reported yet. The chemically modified areca nut husk ash is used for the transesterification of waste cooking oil^21^. The primary reason for hydrothermal carbonization is the low temperature for the sulfonation and reaction medium. To explore the novel potential in one step of the biodiesel manufacturing process, areca nut husk used as a catalyst in the esterification of oleic acid with methanol to produce biodiesel. The parameters like temperature, reactant ratio, and time were investigated for biodiesel conversion. These breakthroughs offer more efficient and cost-effective catalyst manufacturing by adding –SO_3_H groups and retaining feebly acidic hydrophilic groups that can confiscate water and enhance esterification. The as-synthesized catalyst shows high effectiveness for biodiesel production with an efficiency of 96.4%. The catalyst reusability study shows the high stability of catalyst  up to four cycles. Additionally, SANH manufactured using *Areca nut husk* is a waste bio-resources that lowers the cost of FAME production.

## Materials and experimental methods

### Materials used

*Areca nut husk* was acquired from the campus of NIT Silchar, India. OA having purity ≥ 99% was acquired from Sigma Aldrich. BaCl_2_ (99%), MeOH (99%), and H_2_SO_4_ (98%) were purchased from Merck. Deionized water was taken from UV water purification system (Merck). No additional purification was performed on any of the compounds before they were employed.

### Fabrication of catalyst

The husk obtained from the areca nut is washed and dried. Two batch experiments are used for the production of the desired catalyst. The first batch is prepared using a 1:10 ratio of *Areca nut husk* and H_2_SO_4_. And the second batch is synthesized using a 1:20 ratio by hydrothermal carbonization method. Both batches are prepared using 80 °C and 100 °C temperatures. The obtained materials are washed with deionized water until no residues of sulfate appear. The obtained black solid material is dried in the oven overnight. The obtained materials are named SANH18, SANH 28, SANH110, and SANH210. Among all these catalysts, the SANH18 catalyst is used due to highly active sulfonic sites.

### Catalyst characterization

Many techniques are applied to characterize the as-synthesized catalyst for elemental and chemical composition. The KBr pellets were examined using a Bruker 3000 Hyperion Microscope equipped with a Vertex 80 FTIR instrument. Powder XRD having Cu-Kα radiation (2θ = 10–90) and a scan speed of 2° min^–1^ was performed on a Phillips X'pert Pro MPD (multi-purpose diffractometer). SEM–EDS-Mapping (magnification of 10^5×^) was performed on FEI Quanta FEG 200F with Schottky emitter (− 200 V to 30 kV). Gold nanoparticles suspended on carbon substrate while serving HRSEM. Before measuring BET and N_2_ adsorption–desorption using a QuantaChrome Nova 2200e Pore Size and Surface Area Analyzer, the material was degassed at 80 °C for 6 h. CHNS analyzer was used to calculate the sulfur content with the help of Elemental Vario EL III. X-ray photoelectron spectroscopy of the sample was examined on PHI 5000 Versaprobe III with dual-beam charge neutralization. To measure the elements C_60_ ion gun and argon ion gun and the diameter of sample holders are 25 mm and 60 mm. TGA was measured between 30 and 700 °C in the presence of N_2_ gas. The particle size of the material is tested by Zetasizer nano ZSP (ZEN 5600). Joel/JEM 2100 was used for high-resolution transmission electron microscopy (HRTEM) using an electron gun made of LaB6 operating at 200 kV. The acid group density in the catalyst is measured using the acid–base back titration method^22^. The catalyst is mixed with 50 mL of NaOH (0.1 M) solution and stirred for 24 h. The solution obtained after stirring is filtrated and recovered. Then the solution is titrated against NaCl (0.01 M). In this method, phenolphthalein act as an indicator.

### Catalyst activity

Homogeneous methanol: OA (10:1–30:1 M ratio) and heterogeneous catalyst (3–9 wt% OA) were added to an ACE pressure tube and heated to 60–100 °C in a microwave for 40–100 min. Rapid and uniform heating of the reaction mixture under microwave radiation has given microwave-assisted biodiesel manufacturing a boost in popularity. The response rates were considerably increased while reaction times were reduced; as a result, this was both economically and environmentally beneficial. This is done by using thin-layer chromatography to monitor the reaction's development. A rotating vacuum evaporator was used to extract the excess MeOH from the solution.

### Biodiesel characterization

Using NMR spectroscopy, the esterification product's purity was confirmed and densified. FAME's chemical structure and production were investigated using ^1^H and ^13^C-NMR on a Bruker Avance III series equipped with a frequency of 500 MHz and TMS as a reference standard. GC–MS was used to determine the FAME's chemical makeup. Injectors with split/splitless technology were used in GC on an Agilent model 8890 with polar columns DB-WAX & HP-5 MS UI Agilent column and split/split injectors. In the beginning, the oven temperature was 50 °C, which rose at a pace of 5 °C/min to 350 °C. An Agilent 5977 MSD apparatus with a mass range of 1.6–1050 amu was used for the MS component of the GC–MS experiment.

### Catalyst reusability

After the esterification reaction, the heterogeneous catalyst was filtered using Whatman paper. The catalyst was washed several times with methanol to remove impurities on the surface of the catalyst. The obtained material was dried in a vacuum oven at 80 °C for 4 h. The dried material is used for the next esterification process without any treatment.

## Result and discussion

### Effect of temperature on hydrothermal carbonization

The effect of temperature on carbonization plays a significant impact in catalyst activity. When the temperature of the carbonization process increases, the lignin, cellulose, and hemicelluloses part of the *Areca nut husk* is broken down into numerous polycyclic aromatic hydrocarbons through the process of dehydration condensation. In this manner, a skeletal structure is generated that is advantageous for incorporating the sulfonic acid moieties^23^. But a high temperature causes the expansion of the reticular system and an increase in the carbon content resulting in a decrement in sulfonic acid active site groups^24^. Thus, an appropriate temperature is required for the carbonization process to facilitate the sulfonation process.

### Effect of sulfonation

**Figure 1:**
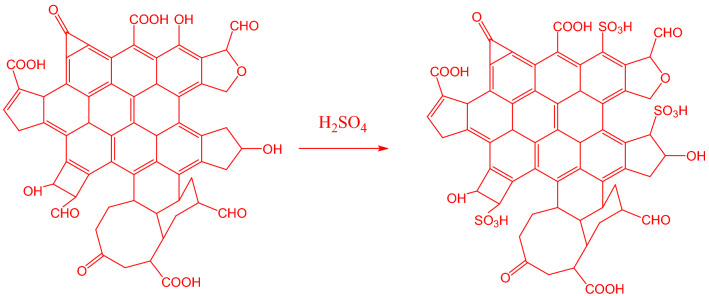
Direct sulfonation of biomass to insert sulfonic active sites.

The temperature had an influence on the activity and stability of the sulfonic acid introduced into biomass. An unstable and readily decomposable chemical was formed as a consequence of the restricted sulfonation. A decrease in catalytic activity may be caused by an unwanted multi-sulfonated group when temperatures rise too high. Increasing the sulfonation temperature to 100 °C and then 120 °C resulted in a lower S-content^25^. However, the degree of functionalization increases with decreasing carbonization temperature; in other words, materials functionalized at lower temperatures are anticipated to have a more significant density of acid sites. At low carbonization temperatures, the biomass structure is not entirely decomposed. As a result, surface groups are formed that may react with sulfuric acid more efficiently, encouraging the increased incorporation of sulfonic groups on the surface of carbon catalysts^24^. Carbon network breaking, as well as high-temperature acid group dehydration, was accountable for the decrease in sulfonation surface areas. The poor thermal resilience of -SO_3_H groups can be attributed to the unfavorable influence on the S content of the catalyst at high reaction temperatures^26^. The maximum S-content measured is of SANH18 catalyst, which owns 3.14% of sulfur. At high H_2_SO_4_ concentrations, the S-content decreases. This is explained by the slower rate of sulfonation-carbonization reactions at higher H_2_SO_4_ concentrations^26^. Figure 1 shows the synthesis of the catalyst by hydrothermal carbonization and the formation of active sites, which are responsible for the esterification process. Table 1 describes the total sulfur present analyzed by CHNS analyzer and the acid density of the synthesized catalysts.

**Table 1:** Sulfur content and acid density of synthesized catalyst.

Catalyst name	Sulfur content (%)	Acid density (mmol g^−1^)
SANH18	3.12	1.88
SANH28	3.05	1.74
SANH110	2.34	1.35
SANH210	2.27	1.27

### FTIR

**Figure 2:**
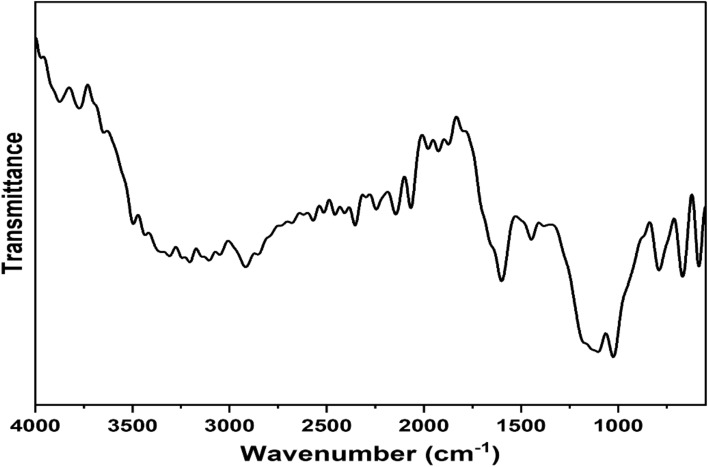
FTIR spectrum of SANH18 catalyst.

FTIR spectrum of the SANH18 catalyst is shown in Fig. 2. Numerous sulfonic acid, hydroxyl, and carbonyl functional groups should be present in an efficient and effective heterogeneous acid catalyst associated with carbonaceous material. FTIR spectrum is used to analyze weak or strong acidic groups, which act as noteworthy active sites for esterification reactions by synergistic effect. The high intensity of the 1028 cm^–1^ peak in the SANH18 catalyst corresponds to the symmetric stretching of –SO_3_H, indicating the formation of a sulfonated catalyst^27^. Our results show the successful sulfonation of biomass-based catalysts even at low temperatures. Zhang et al. claim that the –SO_3_H group replaces hydrogen on the surface of a solid and attaches covalently to carbon^28^. The spectra revealed that the C=C and carbonyl stretching in the aromatic rings were responsible for the 1605 cm^–1^ and 1656 cm^–1^ peak. Peaks 2853–3246 cm^–1^ pertaining to the aldehydes and sp^3^ C–H groups. In the 3359–3756 cm^–1^ region, the O–H stretching modes are clearly visible^29^. This indicates that along with the strong –SO_3_H group, there is the existence of week –OH and –COOH groups.

### XRD

**Figure 3:**
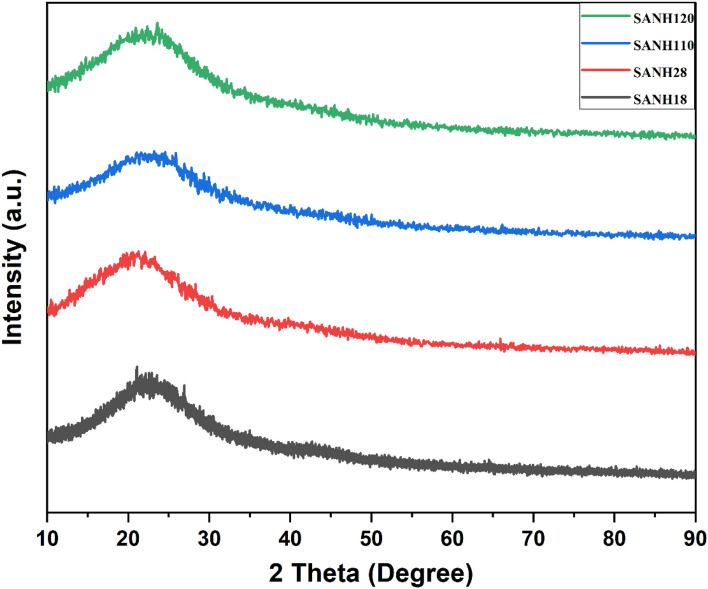
XRD pattern of SANH18 catalyst.

Figure 3 shows the appropriate XRD spectrum of as-fabricated SANH18 catalysts with the greatest sulfur concentration. The catalyst has a wide peak at 2θ = 18–28°, indicating its amorphous nature, in which carbon are positioned arbitrarily^30^. In the SANH18 catalysts, the amorphous phase displays a wider amorphous hump. Polycyclic aromatic carbon sheets are bounded by randomly arranged –SO_3_H, –OH and –COOH groups^31^.

### Morphological and elemental composition analysis

**Figure 4:**
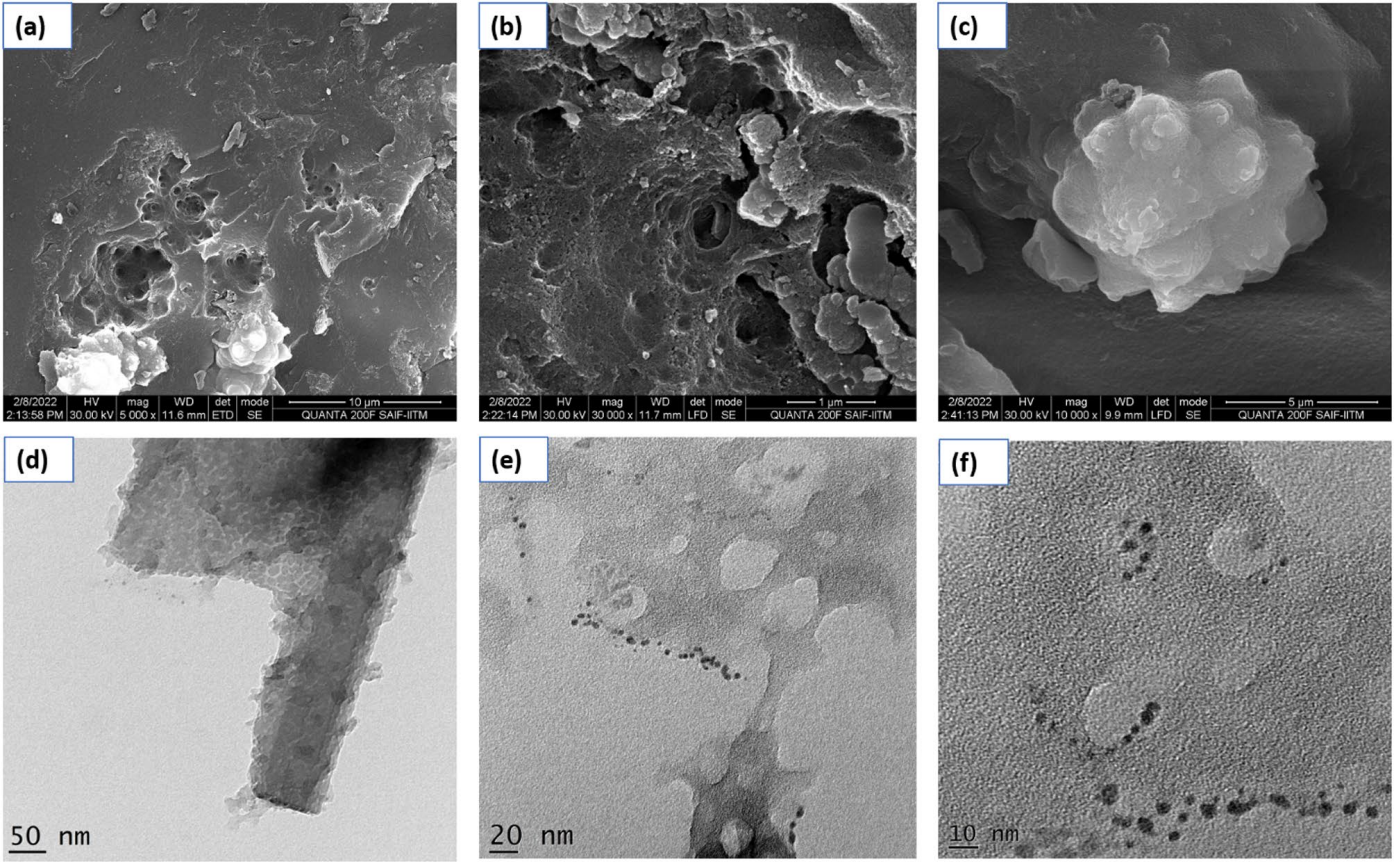
SEM and TEM of catalyst at various resolution.

**Figure 5:**
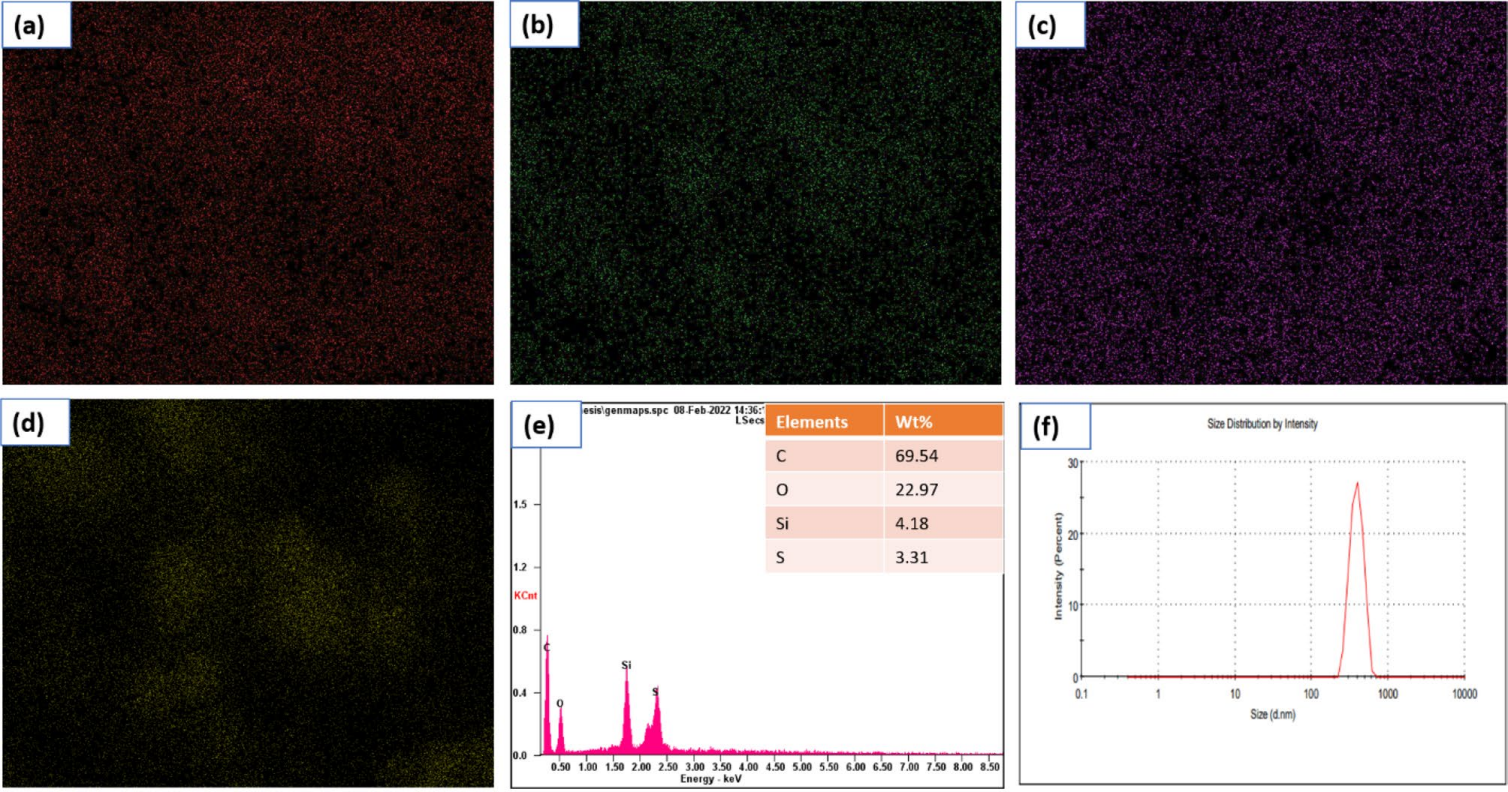
Mapping of the SANH18 catalyst showing (**a**) carbon, (**b**) oxygen, (**c**) sulfur, (**d**) silicon, (**e**) EDS analysis shows the presence of C, O, Si, and S, (**f**) particle size analyzer.

To study the morphology of SANH18 catalyst, SEM and TEM micrographs are taken. The SEM images show the mesoporous surface of the SANH18 catalyst, which is aggregated with different elements which consist of varying particle sizes (Fig. 4a–c). The carbonaceous sheetlike frameworks were detected by TEM (Fig. 4d–f) in the SANH18 catalyst; however, their formation was incomplete^27^. TEM images show that carbonaceous materials exist in the aromatic sheets. Particle size analysis data indicates that the material size ranges in micrometers which is consistent with the TEM results. From the result, it was found that the particle size of the material ranges between 220 to 712 nm. The highest peak of 391.9 nm was observed for the carbonaceous material (Fig. 5f).

Figure 5e depicts the EDS analysis used to identify the components in the catalyst, and it reveals that carbon (73.72%), oxygen (22.97%), and sulfur (3.31%) are present. The confirmation of high sulfur was closely associated with CHNS analyzer as shown in Table 1. Higher sulfur content in SANH18 was shown to have outstanding catalytic activity in the current investigation. In the form of sulfonic acid groups, sulfur was the principal active ingredient, contributing to the performance and activities of the fabricated catalysts. Covalent bonds between sulfuric acid and the catalyst result in greater acid capacity. Mapping of the catalyst shows a close association between C, O, Si, and S and is clearly homogenized and evenly distributed after the extraction (Fig. 5a–d).

Further evidence of the presence of the active species on the catalyst surface was provided by the homogeneous dispersion of sulfur elements. SANH18 had the most incredible sulfur content because of its wide specific surface area and high content of sulfonating agent, both of which contributed to its high sulfur loading. Due to a more considerable extent in the augmentation of surface area and pore volume induced by side reactions such as oxidation, the vast proportion of sulfonating agents resulted in an improved structure of the carbon framework^32^.

### BET

**Figure 6:**
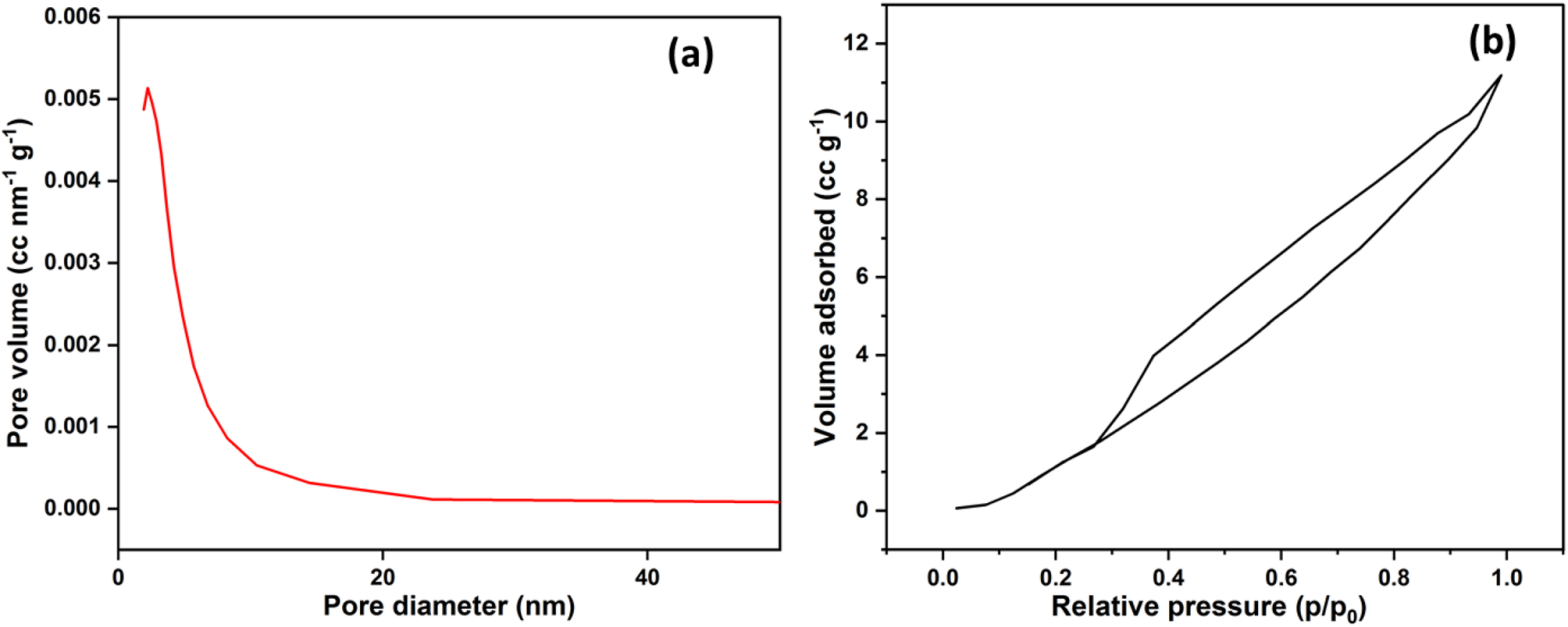
(**a**) BET and (**b**) N_2_ adsorption–desorption curve of synthesized catalyst.

To further understand the surface of SANH18 catalyst, pore size distribution and N_2_ adsorption–desorption isotherm were conducted (Fig. 6a,b). The N_2_ isotherm curve, as in Fig. 6b graphical representation, shows the Type-IV hysteresis loop followed the mesoporous surface as no pore opened at both ends^33^. The data of N_2_ adsorption–desorption clearly match the mesoporous surface (d = 2–50 nm) with the SEM images^34^. To find the pore structure, the BET technique was used to measure the pores' diameter, volume, and surface area. The BET method observed a diameter of 2.21 nm, pore volume of 0.023 cc/g, and surface area of 19.561 m^2^/g. It wasn't only pore shape and surface area that affected esterification reaction catalytic activity. Faster reaction rates may be achieved using mesoporous materials, allowing the reactants to spread into the pores. Whereas in a microporous surface, the reaction occurs at the opening of the pores, resulting in lower reaction rates^35^.

### XPS

**Figure 7:**
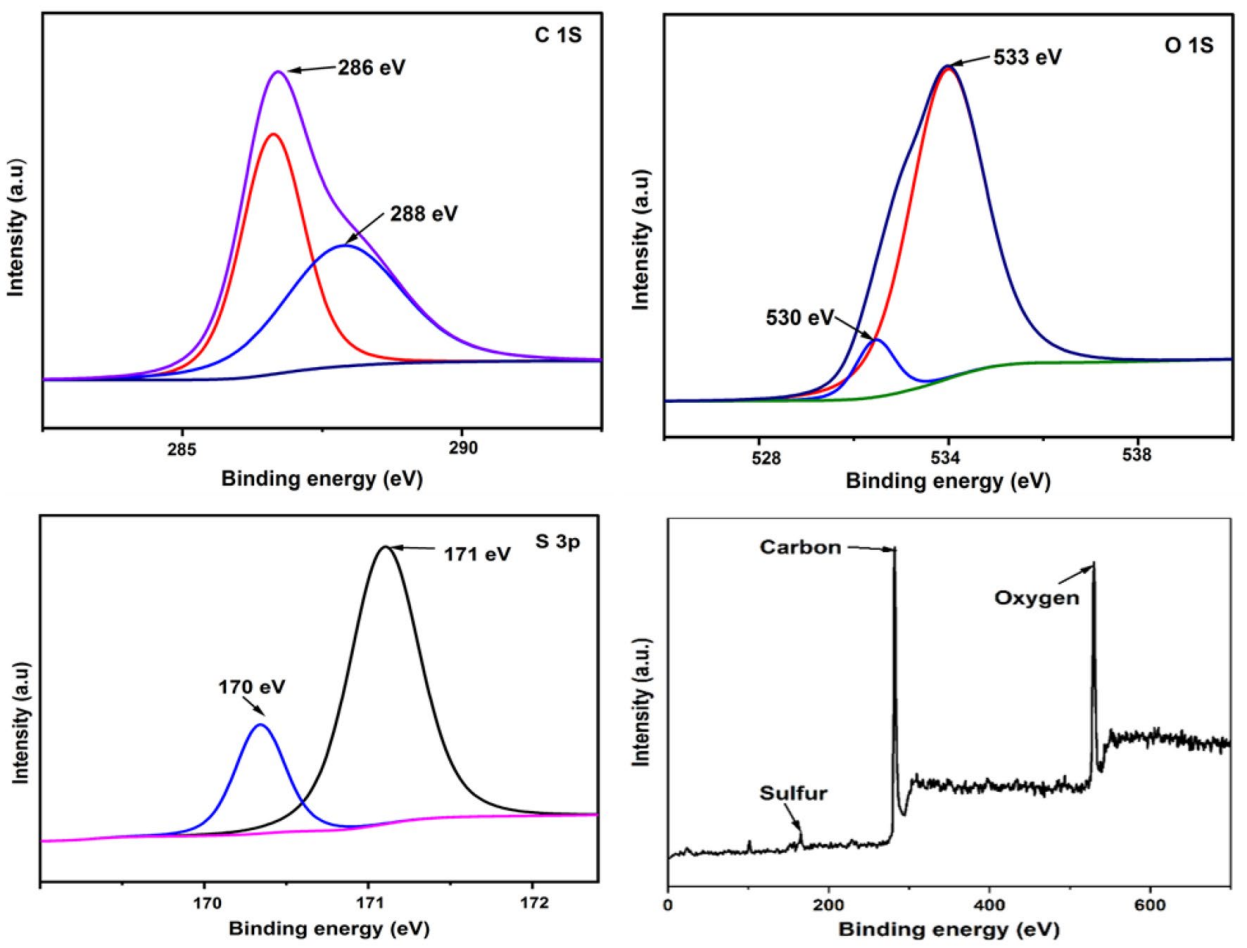
XPS deconvoluted spectra of carbon, oxygen, sulfur and overall spectra.

The chemical valences of the interface group on SANH18 were investigated using XPS characterization. XPS study confirms the presence of carbon, oxygen, and sulfur. The finding of XPS is consistent with EDS analysis (Fig. 5e), indicating the successful sulfonation of biomass at low temperatures. The data of carbon, oxygen, and sulfur is shown in Fig. 7. At 286 and 288 eV, the C_1s_ spectra displayed two peaks. Carbon atoms bonded with sulfur in sulfonic moieties may be allocated to the prominent peak positioned at 286 eV^36^. A C–C and a C=O link in carboxylic groups, both of which have peaks at 286 and 288 eV spectral energies, further demonstrate the existence of oxygen groups^37,38^.

Moreover, the peak in the oxygen spectrum at 531 and 533 eV is ascribed to C–OH and C=O bond^39^. Notably, the S_2p_ area exhibited two peaks at 170 and 171 eV, was ascribed to a high oxidation state of sulfur in -SO_3_H, which is in accordance with FTIR data. The findings from the XPS experiment make it abundantly evident that SANH18 was successfully sulfonated, and they lend credence to the theory that any visible sulfur in the catalyst is present as sulfonic acid groups.

### Thermogravimetric analysis

**Figure 8:**
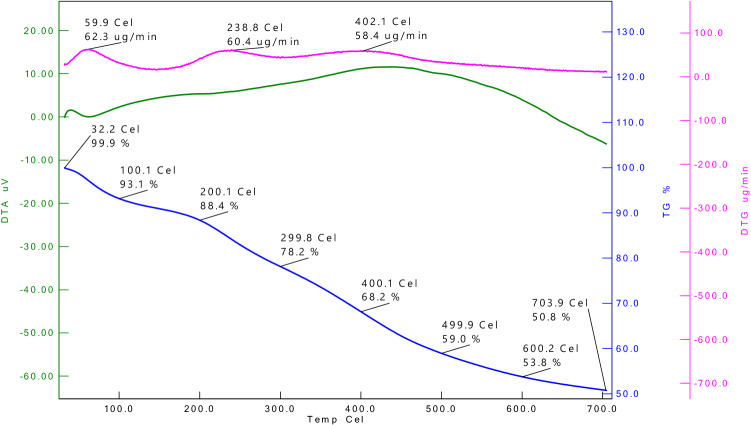
TGA/DTA curve of SANH18 catalyst.

TGA analysis was used to assess the SANH18 catalyst's thermostability. Figure 8 showing TGA thermographs. The primary mass loss of 11.4% SANH18 catalyst up to 200 °C was attributed absorbed water and other volatile substances. In the next phase of 200 °C to 500 °C, the degradation of organic substances occurs such as proteins, lipids and carbohydrates^40^. As a result, a highest mass loss of 29.4% was observed followed by a mass loss of 8.2% at 700 °C due to carbonaceous materials decomposition and releasing CO_2_ and CO etc.^41,42^. Some studies indicate that materials decomposition starts from 420 °C and release CO_2_ and CO^43^.

## Esterification reactions

Esterification reactions were carried out utilizing methanol and oleic acid as well as a catalyst based on sulfuric acid. The study was conducted using a 15 mL glass-sealed tube at 80 °C for 1 h. For esterification reactions, a molar ratio of methanol and oleic acid (15:1, 20:1, 25:1, 30:1) is analyzed. It is clear that as the sulfonating agent concentration was increased, the FAME production also climbed. This is due to the additional sulfonic acid groups that have been integrated into the catalyst surface, giving it enhanced catalytic activity for the esterification step. The FAME conversion was 96.4%, indicating that the synthesized catalysts had promising catalytic activity in the production of biodiesel.

**Figure 9:**
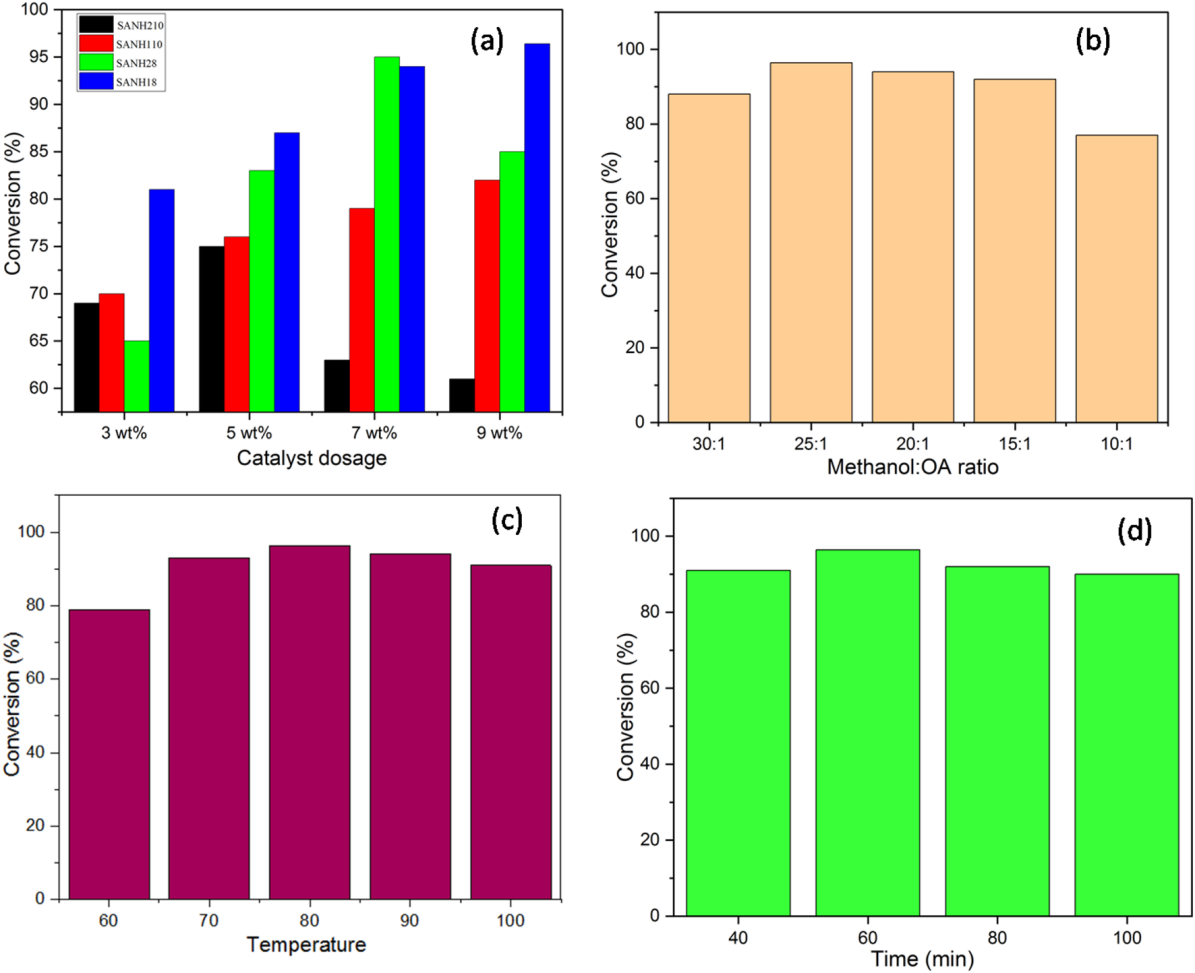
Effect of various parameters on OA/Methanol ratio (**a**) different dosage of *Areca nut husk* catalyst (**b**) methanol/OA ratio (**c**) temperature (**d**) time.

The catalyst dose has a significant impact on the yield of the generation of biodiesel and the rate of reaction. With a catalyst dose of 9 wt%, 96.4% of biodiesel production was achieved, indicating a steep slope in the outcomes (Fig. 9a). Beyond increasing the catalyst dosage, there was no consistent change in the FAME production, indicating that greater doses had no advantages over lower dosages due to increased viscosity and catalytic site saturation^44,45^. As a result, more active sites were accessible to complete the catalytic reaction. As a result, the ideal catalyst dose was determined to be 9 wt%. When contrasted with other acidic catalysts, the synthesized SANH18 demonstrated high efficacy with reduced catalyst loading (9 wt%)^46–49^.

The transformation of the feedstock into FAME may be influenced by the molar ratio of oleic acid to methanol. Figure 9b shows that when the stoichiometric ratio was adjusted from 10 to 30, a substantial improvement was realized. At a molar ratio of 25, the most significant FAMEs yield (96.4%) was achieved (Fig. 9b). However, when the molar ratio was increased to 30, the conversion decreased somewhat. Lower conversion may be caused by partial blocking of the catalyst's active sites due to an overabundance of methanol^50^. The esterification reactions are reversible in nature, so additional alkyl donors are required with respect to oleic acid to reach a conclusion. The reverse reaction can also be decreased due to excess methanol. It was thus decided to proceed to the following optimization phase with a molar ratio of 25. As the molar ratio of methanol to oleic acid increased, so did the catalyst's availability for interaction with the oleic acid. Because of this, the reaction system had a smaller collision frequency with the catalyst, reducing the mass transfer effect^51^.

Temperature is crucial for studying FAME production because it helps reach the activation energy required for the esterification reaction. It is conceivable that an ideal temperature of the reaction must be determined to get a reasonable yield while maintaining a sustainable operating cost. For biodiesel production, temperatures ranged from 60 to 90 °C (Fig. 9c). At the low temperature, the activity was sluggish—the high-temperature results in low viscosity, higher collision frequency, higher diffusion rate, and better solubility. For example, the biodiesel conversion increases as the reaction temperature rise from 70 to 80 °C, reaching 96.4% at 80 °C. The biodiesel output drops to 94.1% at temperatures over 90 °C. To improve interactions and miscibility that lead to bond breaking and cleavages, the reaction temperature makes it easier for molecules of reactants to collide^52^. Uncontrolled vaporization may be to blame for the drop in biodiesel output at 90 °C, which reduces the amount of accessible methanol and, thus, the number of reactive species required for the esterification process^53,54^.

Time is an essential factor in biodiesel synthesis since it affects the activity of the catalyst^55^. As described in Fig. 9d, the time is varied from 40 to 100 min for biodiesel production. The optimum biodiesel conversion was obtained in just 60 min. On further increments of time, there is little change in the conversion of biodiesel. This may be explained by the bidirectional esterification process, which can be induced by prolonging the response time beyond its excellent value. It then ended in the hydrolysis of the biodiesel generated^56^. According to research, a lengthy response time might reduce the surface area by lowering the active sites^57,58^. In 60 min, the maximum amount of biodiesel conversion was obtained (96.4%) and showed the complete utilization of oleic acid. Table 2 summarizes the various parameter effects on the esterification process.

**Table 2:** Effect of various parameter on esterification reaction.

Catalyst loading	Molar ratio OA: MeOH	Temperature (°C)	Time (min)	Efficiency (%)
**Catalyst dosage**				
3 wt%	1:20	80	60	81.2
5 wt%	1:20	80	60	87.6
7 wt%	1:20	80	60	94.3
9 wt%	1:20	80	60	95.7
**Molar ratio of OA and methanol**				
9 wt%	1:10	80	60	77.8
9 wt%	1:15	80	60	92.5
9 wt%	1:20	80	60	95.7
9 wt%	1:25	80	60	96.4
9 wt%	1:30	80	60	88.3
**Temperature**				
9 wt%	1:25	60	60	79.3
9 wt%	1:25	70	60	93.6
9 wt%	1:25	80	60	96.4
9 wt%	1:25	90	60	94.1
9 wt%	1:25	100	60	91.7
**Time**				
9 wt%	1:25	80	40	91.5
9 wt%	1:25	80	60	96.4
9 wt%	1:25	80	80	92
9 wt%	1:25	80	100	90.8

## Kinetic analysis

**Figure 10:**
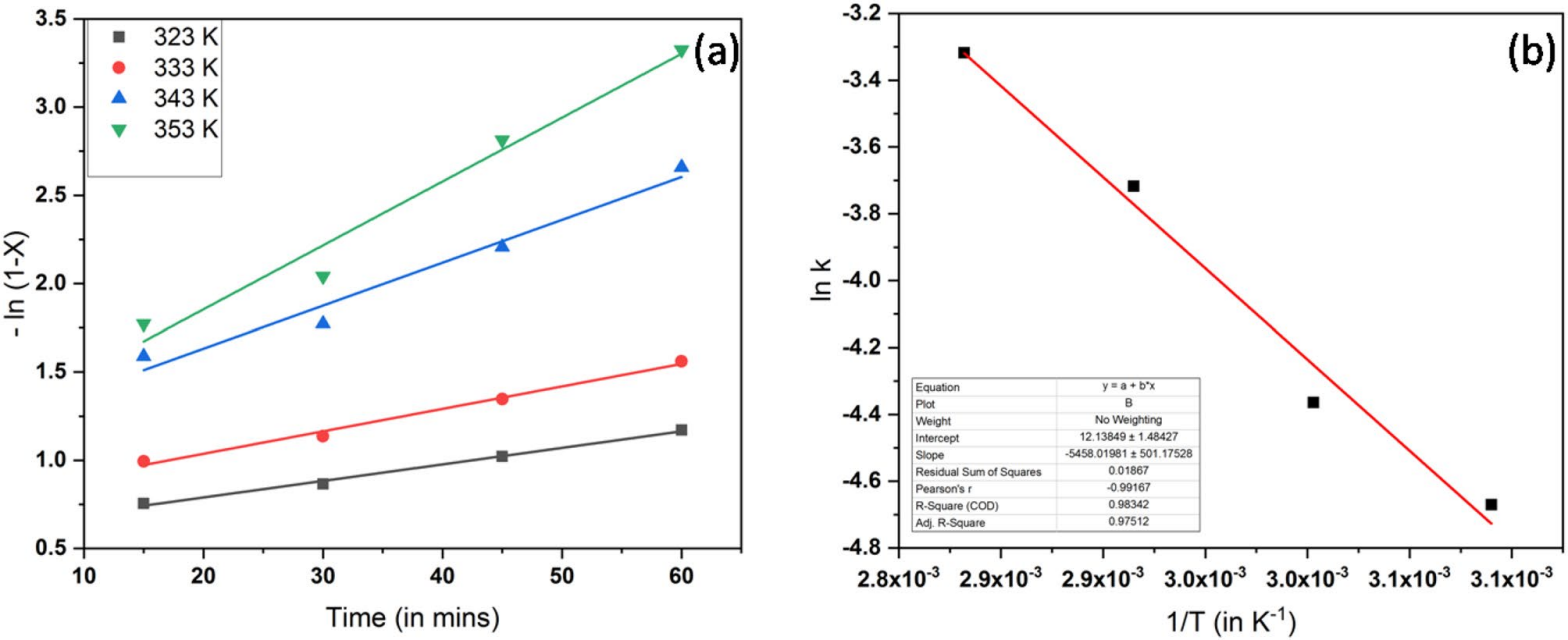
Kinetic study of the reaction (**a**) reaction rate (**b**) Arrhenius plot.

A high concentration of methanol in the esterification reaction postulates the pseudo-first-order reaction^59^. The oleic acid methanolysis reaction takes place in the homogeneous regime by SANH18 catalyst, where the overall reaction rate is determined by chemical reactions. The perceptible rate constant of reaction (k) was determined from the slope of –ln(1−x) vs. reaction time. The obtained linear line indicates the high value of R^2^ (0.97–0.99) confirms the pseudo-first-order reaction^60^ and is used for elucidation of the kinetics of SANH18 (Fig. 10a).

The activation energy was calculated using the Arrhenius equation (Eq. 1) and rate constants at various temperatures (50–80 °C).

1 $${\text{ln k }} = \, {-}{\text{E}}_{{\text{a}}} /{\text{RT }} + {\text{ ln A}}$$

Here, Ea is the activation energy, T is the reaction temperature, A is the Arrhenius constant, and R is the gas constant whose value is 8.314 JK^−1^ mol^−1^. From Fig. 10b, Ea is 45.377 kJ mol^−1^. The range of activation energy lies within the range of 24.7–84.1 kJ mol^−1^, which is suitable for esterification reactions^61^. The as-synthesized catalyst much decreased the activation energy than most of the previous catalysts, H_3_PW_12_O_14_ (51 kJ mol^−1^)^62^, Al-Sr nanocatalyst (72.9 kJ mol^−1^)^63^, and CaO/SiO_2_ (66.3 kJ mol^−1^)^61^.

## Product analysis by GC–MS and NMR

The confirmation of biodiesel production was done by GC–MS, ^1^H-NMR, and ^13^C-NMR. The highest biodiesel production (96.4%) was obtained under the optimization process. The conversion yield was calculated by the area ratio of different esters, the results match with previous study^27^ (Fig. S1). The ^1^H-NMR shows the emergence of characteristics peak at 3.665 ppm corresponding to methoxy protons was compatible with the synthesis of methyl esters (Fig. S2). The further confirmation of methyl ester formation is done with the help of ^13^C-NMR, which shows a signal at 51.435 ppm indicates the formation of methyl esters (Fig. S3). Equation (2) is used to calculate the percentage of conversion yield of biodiesel.

2 $$\mathrm{Biodiesel \; yield }=\frac{Weight \; of \; FAME}{Weight \; of \;OA} \times 100$$

## Catalyst stability and reusability

**Figure 11:**
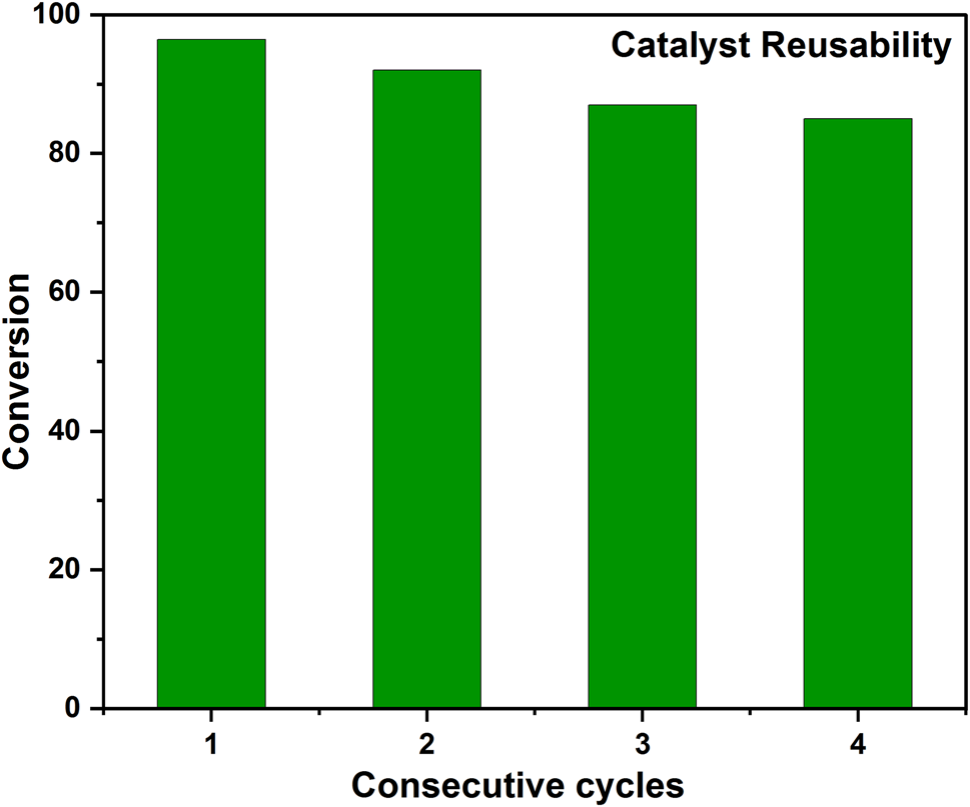
Reusability of SANH18 catalyst.

A suitable catalyst shows high catalytic activity and stability even after several uses. To check the reusability of the SANH18 catalyst, we successfully ran a batch esterification reaction of 1 h in the microwave under optimized conditions (25:1 methanol/OA ratio, 80 °C, 9 wt% catalyst). The catalyst used was washed with methanol with the help of filtration. The subsequent cycle of 1st, 2nd, 3rd, and 4th shows excellent free fatty acid (FFA) conversion of 96.4%, 92%, 87%, and 85%, respectively, as shown in Fig. 11. But after 4 consecutive cycles, the transformation of FFA drops to 75%. According to the literature, this may be due to hydrocarbon residue on the surface of the catalyst, byproducts of the reaction, water adsorption, and reduction in active sulfonic sites^64^. As water is also a byproduct of esterification reaction, so it reduces the activity by acid site contaminations. In the majority of the literature survey, this may be due to the deactivation of catalyst by leaching of SO_3_H groups^27,65^. EDS spectra (Fig. S4b) of reused catalyst reveals the decrease in wt% of sulfur (3.31 wt% to 2.17 wt%). FTIR data (Fig. S4a) of reused also reveals the involvement of SO_3_H groups as the slight peak shifts of these sulfonic groups.

## Conclusion

The batch reactions of solid acid catalyst based on *Areca nut husk* as raw material was synthesized using a one-pot hydrothermal carbonization method at just 80 °C. The sulfuric acid concentration and temperature have a significant effect on the acid density of the SANH18 catalyst. Due to sulfuric acid strength, it causes the release of H^+^ ions, allowing fatty acids carboxylic moieties to be protonated. The carbonaceous catalysts possess high sulfonic groups, performed exceptionally well in the esterification process. The fine-tuned parameters provided better textural qualities regarding total acidity and specific surface area. The maximum esterification by OA was found to be 96.4% under the optimization process (9 wt% catalyst dosage, 25:1 methanol: OA ratio, 80 °C) after 1 h, which is excellent compared to other studies. This stipulates that this acid catalyst is used in many acid-functionalized reactions. The kinetic analysis of the reaction follows the pseudo-first-order due to the high concentration of methanol. The SANH18 catalyst, in the presence of SO_3_H active species, shows perfect activity for the esterification process. The sulfonated catalyst offers high reusability up to four cycles. Moreover, the conversion of FFA into FAME decreases during the reusability test due to humins deposition on the catalyst surface. As a result, the heterogeneous SANH18 catalyst is an effective material for producing economically and environmentally friendly biodiesel. As areca nut husk is found in large quantities in the region, all of the husks go to waste. The catalyst obtained from areca nut husk has a lot of potential to solve environmental problems and is used for sustainable fuel synthesis. The catalyst is particularly successful in early scale-up operations since it is made from a low-cost, easily accessible, and readily renewable kind of biomass, and it also lowers the cost of biodiesel production at an industrial scale.

## Supplementary Information

Supplementary Figures.

## Data Availability

The authors declare that all data supporting the findings of this study are available within the article and its supplementary information files.
