# Supplementary material for: Microwave-assisted synthesis of biodiesel by a green carbon-based heterogeneous catalyst derived from areca nut husk by one-pot hydrothermal carbonization
Source: Sci Rep. 2022 Dec 12;12:21455. doi: 10.1038/s41598-022-25877-w (PMC9744914; doi:10.1038/s41598-022-25877-w)
Supplement: Supplementary file 1 — Supplementary Figures. [file 41598_2022_25877_MOESM1_ESM.docx]

**Microwave-assisted synthesis of biodiesel by a green carbon-based heterogeneous catalyst derived from areca nut husk by one-pot hydrothermal carbonization**

**Gaurav Yadav, Nidhi Yadav, and Md Ahmaruzzaman**

Department of Chemistry National Institute of Technology Silchar, Assam -788010

Supplementary file

Figure S1: GC-MS data

Figure S2: 1H-NMR data

Figure S3: 13C-NMR

Figure S4: Reused FTIR and EDS spectra


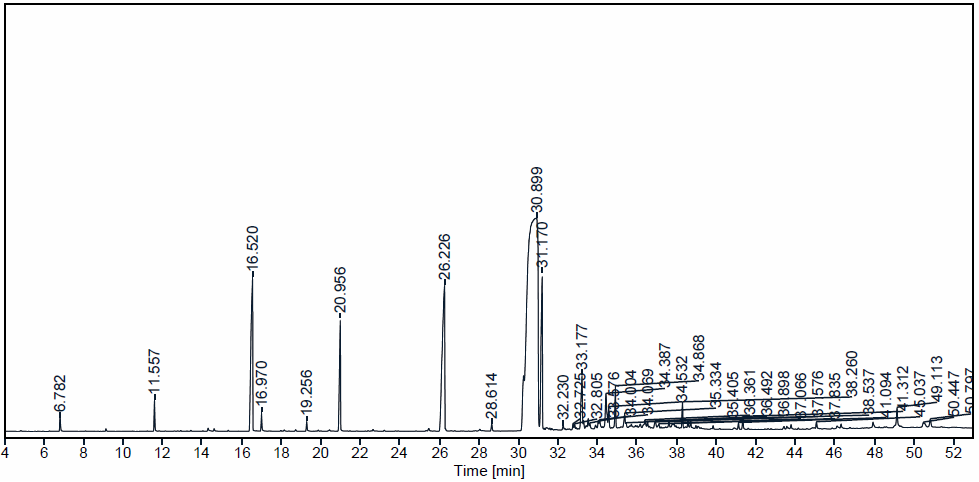


Figure S1: GC-MS data of formed biodiesel. The major compounds formed in the process are methyl oleate (55.10), methyl dodecanoate (8.86%), methyl palmitate (11.48%), and methyl strearate (6.74%).


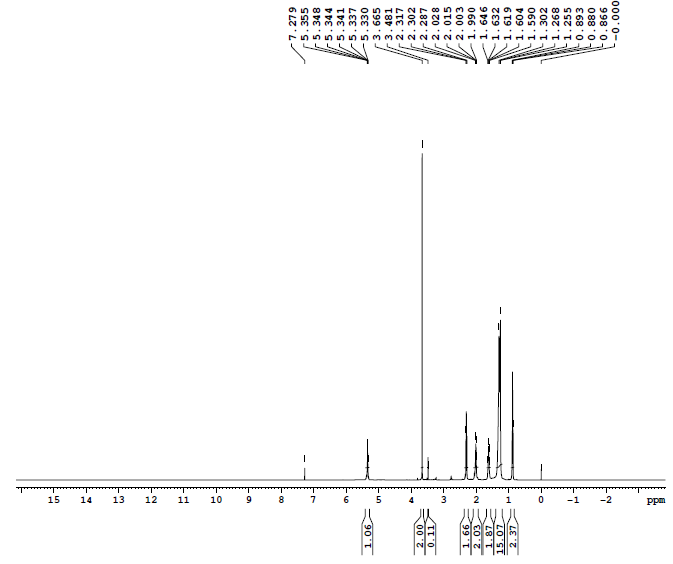


Figure S2: ^1^H-NMR of formed biodiesel


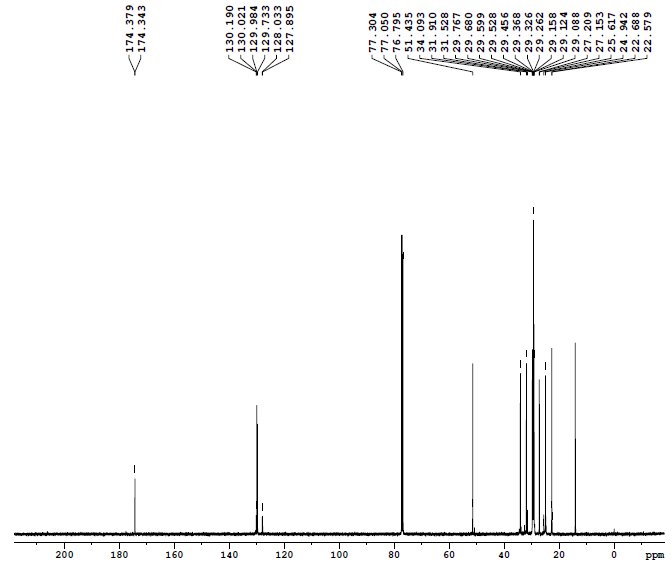


Figure S3: ^13^C-NMR of synthesized biodiesel


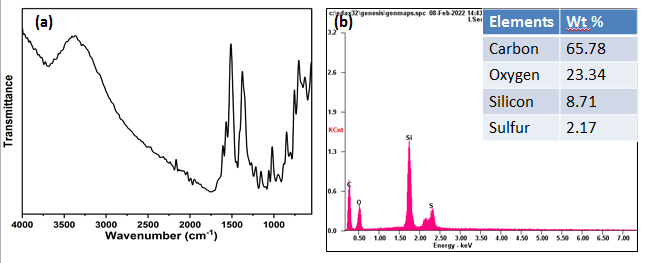


Figure S4: Reused SANH18 (a) FTIR spectra (b) EDS spectra
